# TRANSFER TRAUMA AND MAJOR EVENTS AMONG NURSING HOME RESIDENTS BEFORE AND DURING THE COVID-19 PANDEMIC

**DOI:** 10.1093/geroni/igac059.2853

**Published:** 2022-12-20

**Authors:** Ana Montoya, Pil Park, Chiang-Hua Chang, Julie Bynum

**Affiliations:** University of Michigan, Ann Arbor, Michigan, United States; University of Michigan, Ann Arbor, Michigan, United States; University of Michigan, Ann Arbor, Michigan, United States; University of Michigan, Ann Arbor, Michigan, United States

## Abstract

The negative consequences of transfers are known as transfer trauma. Nursing home (NH)-to-NH transfers place long-term NH residents at risk for developing transfer trauma and this risk may have increased during the COVID-19 pandemic in the setting of a state policy that increased the number of residents who transferred between NHs. The objective of this cross-sectional cohort analysis was to assess the incidence of transfer trauma and major events (hospitalization/death/discharges) among long-term NH residents who transferred from one NH to another before and during the COVID-19 pandemic using a composite measure of transfer trauma based on validated scales from Minimum Data Set (MDS) assessments. A total of 750 residents transferred in the pre-COVID cohort and 795 in the COVID cohort were eligible for assessment of transfer trauma and major events. After adjusting for demographic characteristics, residents in the COVID cohort were almost twice more likely to die and almost three times more likely to discharge within 90 days compared to those in the pre-COVID cohort (AOR=1.94, 95%CI [1.15, 3.26] and AOR= 2.86, 95%CI [2.30, 3.56], respectively). Residents in the COVID cohort were less likely to experience transfer trauma compared to those in the pre-COVID cohort. In the during-COVID cohort, 26% of residents had a COVID-19 diagnosis and they were less likely to experience transfer trauma compared to residents without a COVID-19 diagnosis (AOR=0.34, 95%CI [0.23, 0.50]). It is important to note that some residents may have not stayed in the nursing home long enough to assess them for transfer trauma.

